# Supplementary material for: Soloxolone N-3-(Dimethylamino)propylamide Suppresses Tumor Growth and Mitigates Doxorubicin-Induced Hepatotoxicity in RLS40 Lymphosarcoma-Bearing Mice
Source: Int J Mol Sci. 2025 Dec 10;26(24):11912. doi: 10.3390/ijms262411912 (PMC12732862; doi:10.3390/ijms262411912)
Supplement: Supplementary file 1 [file ijms-26-11912-s001.zip › ijms-4002905-supplementary.pdf]

Article

Soloxolone *N*-3-(Dimethylamino)propylamide Suppresses Tumor Growth and Mitigates Doxorubicin-Induced Hepatotoxicity in RLS40 Lymphosarcoma-Bearing Mice

Arseny D. Moralev [1,2,†], Aleksandra V. Sen'kova [1,†], Alina A. Firsova [2,3], Daria E. Solomina [2,3], Artem D. Rogachev [2,3], Oksana V. Salomatina [1,3], Nariman F. Salakhutdinov [3], Marina A. Zenkova [1] and Andrey V. Markov [1,*]

[1] Institute of Chemical Biology and Fundamental Medicine, Siberian Branch of the Russian Academy of Sciences, Lavrent'ev Ave., 8, 630090 Novosibirsk, Russia; arseniimoralev@gmail.com (A.D.M.); senkova_av@1bio.ru (A.V.S.); ana@nioch.nsc.ru (O.V.S.); marzen@niboch.nsc.ru (M.A.Z.)
[2] Faculty of Natural Sciences, Novosibirsk State University, Pirogov St. 2, 630090 Novosibirsk, Russia; alina.okhina@mail.ru (A.A.F.); d.solomina@g.nsu.ru (D.E.S.); rogachev@nioch.nsc.ru (A.D.R.)
[3] N. N. Vorozhtsov Novosibirsk Institute of Organic Chemistry, Siberian Branch of the Russian Academy of Sciences, Lavrent'ev Ave., 9, 630090 Novosibirsk, Russia; anvar@nioch.nsc.ru
* Correspondence: markov_av@1bio.ru; Tel.: +7-383-363-51-61
† These authors contributed equally to this work.

Supplementary Materials

Table S1. Animal body weights

Day after tumor transplantation	Mean body weight, g					
	Healthy	Control	Vehicle	DOX	Sol-DMAP	Sol-DMAP + DOX
Day 4	19.3	19.8	19.9	19.5	19.3	20.4
Day 6	19.3	20.5	20.2	19.1	19.5	19.9
Day 8	19.7	20.6	20	18.8	19.3	19.8
Day 11	20	19.3	20.6	17.9	19.6	18.8
Day 13	20.3	19.9	20.8	17.2	19.7	18.3
Day 14	19.3	19.8	19.6	16.9	19.6	17.5

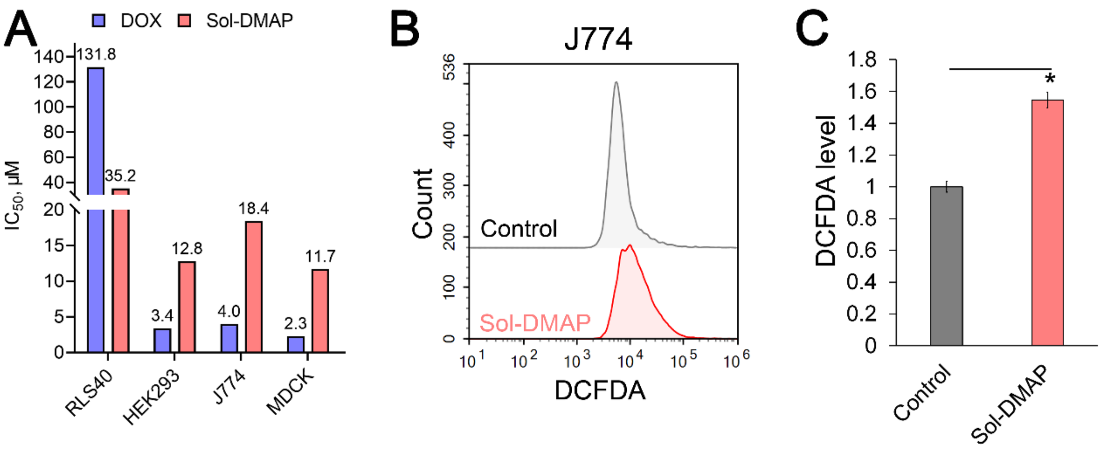

Figure S1. Cytotoxic and ROS induction effects of Sol-DMAP in non-malignant immortalized cells. (**A**) IC_{50} values of Sol-DMAP in RLS40 and non-tumor cells (HEK293, J774, MDCK). (**B**, **C**) ROS production in J774 cells after 24 h of incubation with Sol-DMAP (18 μM) assessed by flow cytometry after DCFDA staining. Representative histogram (**B**) and relative DCFDA level (**C**).

Table S2. Mass spectrometry parameters for detection of Sol-DMAP, DOX and 2-Ad in MRM

Analyte and its parent ion m/z, Da (Q1)	Fragment ion m/z, Da (Q3)	Dwell time. ms	DP, V	EP, V	CE, V	CXP, V
Sol-DMAP (576.1)	91.1	50	96	9	129	4
	531.3	50	96	11	61	6
	516.3	50	91	11.5	47	6
DOX (544.1)	397.1	50	226	10	15	26
	361.1	50	226	10	35	26
	321.0	50	226	10	35	20
	346.0	50	226	10	55	24
2-Ad (152.2)	93.1	50	16	10	35	14
	107.2	50	21	10	37	8

Table S3. List of primers and probes used in the study.

Organism	Gene Symbol	Accession	Type	Sequences of Primers and Probes, 5′ → 3′	Amplicon length	Exon	Primer, nM	Efficiency, %
Human	*HMOX1*	NM_002133.3	Forward	AGAATGCTGAGTTCATGAGGA	90	2/3	500	94.81
			Reverse	CATAGATGTGGTACAGGGAGG			500	
	GCLC	NM_001498.4	Forward	GGGGCGATGAGGTGGAAT	172	1/3	500	96.40
			Reverse	CCTTCAATCATGTAACTCCCATAC			500	
	GCLM	NM_002061.4	Forward	ATCAAACTCTTCATCATCAAC	116	3/4	500	98.56
			Reverse	GATTAACTCCATCTTCAATAGG			500	
	NQO1	NM_000903.3	Forward	TGAAGAAGAAAGGATGGGAGG	223	2/3	500	93.45
			Reverse	AGGGGGAACTGGAATATCAC			500	
	HPRT1	NM_000194.3	Forward	TATGGCGACCCGCAGCCCT	158	1/3	500	97.03
			Reverse	CATCTCGAGCAAGACGTTCAG			500	
Mice	*Tbp*	NM_013684.3	Forward	CACCAATGACTCCTATGACCC	114	3/4	500	103.56
			Probe	((5,6)-ROX)-CACTCCTGCCACACCAGCTTCT-BHQ2			500	
			Reverse	CAAGTTTACAGCCAAGATTCACG			500	
	Mdr1b	NM_011075.2	Forward	CGAAAGCCAAAGTATCAGCATC	148	24/25	500	93.41
			Probe	((5,6)-FAM)-5'-ATTGACAGCTACAGCACAGAGGGC-3'-BHQ1			500	
			Reverse	GGTCGGGTGGGATAGTTAAAC			500	
